# Association of Major Depression With Risk of Ischemic Heart Disease in a Mega‐Cohort of Chinese Adults: The China Kadoorie Biobank Study

**DOI:** 10.1161/JAHA.116.004687

**Published:** 2016-12-21

**Authors:** Na Liu, Xiong‐Fei Pan, Canqing Yu, Jun Lv, Yu Guo, Zheng Bian, Ling Yang, Yiping Chen, Tangchun Wu, Zhengming Chen, An Pan, Liming Li, the China Kadoorie Biobank Collaborative Group, Junshi Chen, Rory Collins, Richard Peto, Garry Lancaster, Xiaoming Yang, Alex Williams, Margaret Smith, Yumei Chang, Guoqing Zhao, Lixue Wu, Can Hou, Zengchang Pang, Shaojie Wang, Yun Zhang, Kui Zhang, Silu Liu, Zhonghou Zhao, Shumei Liu, Zhigang Pang, Weijia Feng, Shuling Wu, Liqiu Yang, Huili Han, Hui He, Xianhai Pan, Shanqing Wang, Hongmei Wang, Xinhua Hao, Chunxing Chen, Shuxiong Lin, Xiaoshu Hu, Minghao Zhou, Ming Wu, Yeyuan Wang, Yihe Hu, Liangcai Ma, Renxian Zhou, Guanqun Xu, Baiqing Dong, Naying Chen, Ying Huang, Mingqiang Li, Jinhuai Meng, Zhigao Gan, Jiujiu Xu, Yun Liu, Xianping Wu, Yali Gao, Ningmei Zhang, Guojin Luo, Xiangsan Que, Xiaofang Chen, Pengfei Ge, Jian He, Xiaolan Ren, Hui Zhang, Enke Mao, Guanzhong Li, Zhongxiao Li, Jun He, Guohua Liu, Baoyu Zhu, Gang Zhou, Shixian Feng, Yulian Gao, Tianyou He, Li Jiang, Jianhua Qin, Huarong Sun, Liqun Liu, Min Yu, Yaping Chen, Zhixiang Hu, Jianjin Hu, Yijian Qian, Zhiying Wu, Lingli Chen, Wen Liu, Guangchun Li, Huilin Liu, Xiangquan Long, Youping Xiong, Zhongwen Tan, Xuqiu Xie, Yunfang Peng

**Affiliations:** ^1^ Department of Nutrition and Food HygieneMinistry of Education Key Laboratory of Environment and Health, and State Key Laboratory of Environmental Health (Incubating)School of Public HealthTongji Medical CollegeHuazhong University of Science and TechnologyWuhanChina; ^2^ Department of Epidemiology and BiostatisticsMinistry of Education Key Laboratory of Environment and Health, and State Key Laboratory of Environmental Health (Incubating)School of Public HealthTongji Medical CollegeHuazhong University of Science and TechnologyWuhanChina; ^3^ Department of Occupational and Environmental HealthMinistry of Education Key Laboratory of Environment and Health, and State Key Laboratory of Environmental Health (Incubating)School of Public HealthTongji Medical CollegeHuazhong University of Science and TechnologyWuhanChina; ^4^ Department of Health CareThe First Affiliated Hospital with Nanjing Medical UniversityNanjingChina; ^5^ Department of Epidemiology and BiostatisticsSchool of Public HealthPeking University Health Science CenterBeijingChina; ^6^ Chinese Academy of Medical SciencesBeijingChina; ^7^ Clinical Trial Service Unit & Epidemiological Studies Unit (CTSU)Nuffield Department of Population HealthUniversity of OxfordUnited Kingdom

**Keywords:** Chinese, depression, ischemic heart disease, prospective cohort study, Epidemiology, Mental Health, Coronary Artery Disease, Primary Prevention, Risk Factors

## Abstract

**Background:**

Increasing evidence has suggested that major depression (MD) is associated with an increased risk of ischemic heart disease (IHD). We examined this association in Chinese adults using data from the China Kadoorie Biobank study.

**Methods and Results:**

Over 0.5 million adults aged 30 to 79 years were followed from baseline interview (2004–2008) until December 31, 2013. Past year MD was measured with the modified Chinese version of Composite International Diagnostic Interview‐Short Form at baseline. Incident IHD cases were identified through linkage to related medical databases, and defined as having *International Statistical Classification of Diseases and Related Health Problems* 10th Revision codes of I20 to I25. Cox proportional hazards regression models were used to estimate hazard ratios and 95% CIs for the MD‐IHD association with adjustment for sociodemographic variables and established cardiovascular risk factors. During 3 423 542 person‐years of follow‐up, 24 705 incident IHD cases were documented. Higher IHD incidence was observed in participants with MD compared with those without (8.76 versus 7.21 per 1000 person‐years), and the multivariable‐adjusted hazard ratio was 1.32 (95% CI 1.15–1.53). Geographic location modified the association (*P* for interaction=0.005), and a positive association was observed in urban residents (hazard ratio 1.72; 95% CI 1.39–2.14) but not rural residents (1.13; 0.93–1.37). Compared with participants without depressive symptoms, the hazard ratio (95% CI) of IHD was 1.13 (1.04–1.23) for those with depressive symptoms only and 1.33 (1.15–1.53) for those with MD.

**Conclusions:**

Past year major depression was associated with an increased risk of IHD in Chinese adults, independent of other major cardiovascular risk factors.

## Introduction

Based on the Global Burden of Disease Study 2010, ischemic heart disease (IHD) caused an estimated over 129 million disability‐adjusted life years in 2010, about a 29% increase from 1990.^1^ IHD remained as one of the leading causes of death and disability‐adjusted life years in the Global Burden of Disease Study 2013.^2^ In China, a total of 230 million adults suffered from IHD in 2010, and the number will continue to increase dramatically because of aging, urbanization, and lifestyle changes.^3^ As predicted, the number of IHD patients in Chinese adults aged 35 to 84 years old would increase by 64% during 2020 and 2029.^4^ Therefore, identification of risk factors for IHD and implementation of public health measures to reduce the risk factors in Chinese adults will have a tremendous impact on national and global population health.

Major depression (MD), featuring sadness or irritability accompanied by a cluster of psychophysiological disturbances (such as weight change, sleeping problems, inability to experience pleasure in daily life, difficulty in concentrating, and thoughts of death) can lead to severe impairment in quality of life and physical functioning.^5^ The Global Burden of Disease Study 2013 showed that MD was among the top causes of disability‐adjusted life years and years lived with disability worldwide.^6^ Mounting evidence indicates that MD is related to an increased risk of morbidity and mortality of IHD as reviewed in previous meta‐analyses.^7^, ^8^, ^9^ However, most studies were conducted in Western populations, and few were in Asian or Chinese populations. One study in Chinese adults aged 20 years and older using data from the Taiwan Health Insurance database found a significant increased risk of composite coronary events associated with clinically diagnosed MD,^10^ while another study in Chinese adults aged 65 years or older in Hong Kong found a significant association between self‐reported depressive symptoms and mortality from coronary heart disease (CHD) only in men but not in women.^11^

To the best of our knowledge, no prospective cohort study has been conducted in mainland China to examine the relation of depression with incident IHD. To address the gap, we aim to examine the association between MD and risk of IHD using data from the China Kadoorie Biobank (CKB) study.

## Methods

### Study Population

The CKB study is a prospective population‐based cohort study among adults aged 30 to 79 years from 10 geographically defined regions of China (5 urban and 5 rural). These regions were selected according to disease patterns and risk exposures, economic development, population stability, and other major population characteristics. A detailed description about the CKB study has been published previously.^12^ All participants were interviewed face‐to‐face at baseline by trained staff using a standardized electronic questionnaire that covered demographic characteristics, socioeconomic status, personal behaviors, general health, family history, mental health, and for women only, reproductive history.

A total of 512 891 individuals (210 259 men, 41.0%) were enrolled at the baseline interview (2004–2008). In the current study, 26 350 participants were excluded because of previous diagnosis of cancer (n=2577), CHD (n=15 472), rheumatic heart disease (n=938), stroke or transient ischemic attack (n=8884), and missing value on body mass index (BMI) (n=2), leaving 486 541 participants in the final analysis. Ethical approval has been obtained from the Ethics Review Committee of the Chinese Center for Disease Control and Prevention, Beijing, China, and Oxford Tropical Research Ethics Committee, University of Oxford, Oxford, United Kingdom. Written informed consent was obtained from each study participant.

### Assessment of Major Depression

At baseline, participants were asked whether they had had situations for 2 weeks or more in a row during the past 12 months: (1) feeling much more sad, or depressed than usual; (2) loss of interest in most things like hobbies or activities that usually give you pleasure; (3) felt so hopeless that you had no appetite to eat even your favorite food; and (4) feeling worthless and useless, everything that went wrong was your fault and life was very difficult so that there was no way out. If the answer was “yes” to any of the 4 questions, the participants were further assessed for MD using the modified Chinese version of Composite International Diagnostic Interview‐Short Form in a face‐to‐face interview by trained health workers at study clinics. The Composite International Diagnostic Interview, a fully structured diagnostic instrument, is based on the case definition from the *Diagnostic and Statistical Manual of Mental Disorders IV* and has shown moderate concordance with clinical psychiatric interviews.^13^, ^14^ The validity of the Chinese version has been published elsewhere.^15^ Participants were defined as having past year MD if they had felt sad, blue, or depressed for ≥2 weeks during the past 12 months, and if they had at least 3 of 7 additional symptoms, including loss of interest and pleasure, loss of energy or fatigue, weight change, sleep problems, concentration problems, feelings of worthlessness, and thoughts of suicide. In our current analysis, participants who responded positively to the screening questions but did not fulfill the criteria for MD were categorized as having depressive symptoms only.

### Assessment of IHD

The main outcome of interest was incident IHD, which was defined as categories I20 to I25 according to the *International Statistical Classification of Diseases and Related Health Problems* 10th Revision. In the CKB study, incident IHD was collected by linkage to established disease registries and national health insurance claim databases. The disease registries were currently available in 8 of the 10 study areas, while the national health insurance claim databases covered all study areas and recorded details of all hospitalized episodes including disease characteristics, diagnostic procedures, and *International Statistical Classification of Diseases and Related Health Problems* 10th Revision codes.^12^ The linkage to the health insurance database was renewed annually, and those who failed to be linked were actively followed annually by local research staff to ascertain their status, including hospital admission, death, and migration. Only new IHD cases confirmed by medical records were considered as incident cases.

### Assessment of Covariates

Sociodemographic characteristics, lifestyle factors, and medical history were collected at the baseline interview through a laptop‐based questionnaire. Sociodemographic characteristics included age, sex, education level (categorized as no formal school, primary school, middle school, high school, and college/university or more), annual household income (categorized as less than 5000, 5000–9999, 10 000–19 999, and 20 000 or more, in RMB Yuan), and marital status (categorized as married, widowed, separated/divorced, and never married). Lifestyle factors included smoking (categorized as never, former, occasional and current), alcohol drinking (categorized as less than weekly and weekly or more), and total physical activity (calculated as metabolic equivalent task hours per day [MET‐hours/day] spent on work and leisure activities). Participants were also asked about their personal medical history and family history of major chronic diseases.

Body weight, height, and blood pressure were measured at baseline by trained staff using calibrated instruments according to standardized protocols. BMI was calculated as weight in kilograms divided by height in meters squared. Prevalent hypertension was defined as systolic blood pressure ≥140 mm Hg, diastolic blood pressure ≥90 mm Hg, self‐reported diagnosis of hypertension, or self‐reported use of antihypertensive medication at baseline. Prevalent diabetes mellitus was defined as fasting blood glucose ≥7.0 mmol/L, random blood glucose ≥11.1 mmol/L, self‐reported diagnosis of diabetes mellitus, or use of antidiabetic medications.

### Statistical Analysis

Baseline characteristics were compared according to MD status using Student *t* test for continuous variables and χ^2^ test for categorical variables. Follow‐up time was defined as the time interval between the date of baseline interview and the date of IHD diagnosis, death of any cause, loss to follow‐up, or December 31, 2013, whichever occurred first. The association between baseline MD status and incident IHD was estimated by Cox proportional hazards regression models. We examined the proportional hazards assumptions and no violations were found. We adjusted for sociodemographic factors (including age, sex, geographic location, marital status, education level, and annual household income) in the first model, and additionally adjusted for lifestyle factors (smoking status, drinking status, and physical activity) in the second model. Finally, family history of heart attack, BMI, history of diabetes mellitus, and hypertension were included in the third model.

Stratified analyses were conducted to investigate whether the association between MD and incident IHD varied by prespecified factors, including sex, age, BMI, and geographic location. Effect modification by these factors was assessed by including an interaction term in the final model. To explore whether there was dose–response relationship, we repeated the multivariable analysis using reclassified MD status (no depressive symptoms, depressive symptoms only, and MD). To minimize the possibility of reverse causality, a sensitivity analysis was conducted by removing all incident cases that occurred within the first 2 years of follow‐up (n=5268).

All analyses were performed using SAS software, version 9.2 (SAS Institute). All *P* values were 2 sided, and the level of statistical significance was defined at *P*<0.05.

## Results

The overall prevalence of MD was 0.61% (n=2972) among 486 541 Chinese adults. All differences in baseline characteristics between participants with and without MD were statistically significant (Table 1). Participants who reported MD in the past 12 months were younger and less educated, were more likely to be females, rural residents, never smokers, and light drinkers, and they had lower BMI and were less likely to be physically active. They were also less likely to be married and wealthy. For women, those with MD were more likely to be at their perimenopause period. In addition, those with MD tended to have a family history of heart attack, and suffer from diabetes mellitus but not hypertension.

**Table 1:** Baseline Characteristics of Participants by MD Status^a^

Variables	Overall	Participants With MD	Participants Without MD	*P* Values
N	486 541	2972	483 569	
Age, y	51.0±10.5	50.4±9.9	51.0±10.5	<0.001
Sex				<0.001
Male	199 113	837 (28.2)	198 276 (41.0)	
Female	287 428	2135 (71.8)	285 293 (59.0)	
Menopausal status (women only)				<0.001
Premenopause	127 325	869 (40.7)	126 456 (44.3)	
Perimenopause	14 354	128 (6.0)	14 226 (5.0)	
Postmenopause	145 749	1138 (53.3)	144 611 (50.7)	
Marital status				<0.001
Married	442 228	2220 (74.7)	440 008 (91.0)	
Widowed	33 081	570 (19.2)	32 511 (6.7)	
Separated/divorced	7579	139 (4.7)	7440 (1.5)	
Never married	3653	43 (1.5)	3610 (0.8)	
Education level				<0.001
No formal school	90 829	654 (22.0)	90 175 (18.7)	
Primary school	156 407	1048 (35.3)	155 359 (32.1)	
Middle school	138 361	800 (26.9)	137 561 (28.5)	
High school	73 322	353 (11.9)	72 969 (15.1)	
College/university or more	27 622	117 (3.9)	27 505 (5.7)	
Annual household income, RMB				<0.001
<5000	47 472	565 (19.0)	46 907 (9.7)	
5000 to 9999	90 421	635 (21.4)	89 786 (18.6)	
10 000 to 19 999	140 432	855 (28.8)	139 577 (28.9)	
≥20 000	208 216	917 (30.9)	207 299 (42.9)	
Geographic location				<0.001
Urban	209 786	1001 (33.7)	208 785 (43.2)	
Rural	276 755	1971 (66.3)	274 784 (56.8)	
Smoking status				<0.001
Never	301 601	2054 (69.1)	299 547 (62.0)	
Former	26 807	108 (3.6)	26 699 (5.5)	
Occasional	27 872	171 (5.8)	27 701 (5.7)	
Current	130 261	639 (21.5)	129 622 (26.8)	
Drinking status				<0.001
Less than weekly	412 950	2672 (89.9)	410 278 (84.8)	
Weekly or more	73 591	300 (10.1)	73 291 (15.2)	
Family history of heart attack				<0.005
Yes	13 269	109 (3.7)	13 160 (2.7)	
No	473 272	2863 (96.3)	470 409 (97.3)	
BMI, kg/m^2^	23.6±3.4	23.1±3.4	23.6±3.4	<0.001
Physical activity, MET‐hours/day	21.6±13.9	20.7±14.2	21.6±13.9	0.001
Baseline history of diabetes mellitus				0.025
Yes	26 118	187 (6.3)	25 931 (5.4)	
No	460 423	2785 (93.7)	457 638 (94.6)	
Baseline history of hypertension				<0.001
Yes	158 473	874 (29.4)	157 599 (32.6)	
No	328 068	2098 (70.6)	325 970 (67.4)	

BMI indicates body mass index; MD, major depression; MET, metabolic equivalent task.

a Data are shown as n (%) or mean±SD, unless otherwise specified. *P* values were derived from Student *t* test for continuous variables and χ^2^ test for categorical variables.

A total of 24 705 incident cases of IHD were identified over a median follow‐up of 7.2 years. The incidence rate of IHD was 8.76 per 1000 person‐years among participants with MD versus 7.21 per 1000 person‐years among those without MD (Table 2). The hazard ratio (HR) and 95% CI between baseline MD status and incident IHD was 1.32 (1.14–1.52) after adjustment for sociodemographic factors (Table 2). The positive association remained after further adjustment for lifestyle factors, baseline comorbidities, and family history of heart diseases (HR 1.32, 95% CI 1.15–1.53).

**Table 2:** MD and Risk of Incident Ischemic Heart Disease: Stratified Analysis and Sensitivity Analysis

	Incidence Rate (1000 Person‐Years)	Model 1 HR (95% CI)	Model 2 HR (95% CI)	Model 3 HR (95% CI)	*P* for Interaction^a^
Overall					—
No MD	7.21	1.00	1.00	1.00	
Past year MD	8.76	1.32 (1.14–1.52)	1.30 (1.13–1.50)	1.32 (1.15–1.53)	
Sex					0.23
Men					
No MD	7.34	1.00	1.00	1.00	
Past year MD	9.55	1.53 (1.18–1.98)	1.47 (1.13–1.91)	1.50 (1.15–1.94)	
Women					
No MD	7.12	1.00	1.00	1.00	
Past year MD	8.47	1.25 (1.05–1.48)	1.24 (1.05–1.48)	1.26 (1.07–1.50)	
Age					0.10
<60 years old					
No MD	4.76	1.00	1.00	1.00	
Past year MD	6.93	1.42 (1.19–1.69)	1.39 (1.17–1.66)	1.42 (1.19–1.69)	
≥60 years old					
No MD	16.35	1.00	1.00	1.00	
Past year MD	17.42	1.14 (0.89–1.45)	1.12 (0.88–1.43)	1.15 (0.90–1.46)	
BMI					0.98
<24 kg/m^2^					
No MD	6.26	1.00	1.00	1.00	
Past year MD	7.88	1.33 (1.10–1.61)	1.31 (1.09–1.59)	1.31 (1.08–1.58)	
≥24 kg/m^2^					
No MD	8.46	1.00	1.00	1.00	
Past year MD	10.28	1.34 (1.08–1.66)	1.33 (1.07–1.65)	1.33 (1.07–1.66)	
Geographic location					0.005
Rural					
No MD	6.60	1.00	1.00	1.00	
Past year MD	7.35	1.14 (0.94–1.38)	1.12 (0.92–1.35)	1.13 (0.93–1.37)	
Urban					
No MD	8.03	1.00	1.00	1.00	
Past year MD	11.65	1.72 (1.38–2.13)	1.68 (1.35–2.08)	1.72 (1.39–2.14)	
Excluding cases occurring within the first 2 years					—
No MD	5.68	1.00	1.00	1.00	
Past year MD	6.98	1.29 (1.10–1.51)	1.27 (1.08–1.49)	1.29 (1.10–1.52)	

Model 1: adjusted for age, sex, geographic location, marital status, education, and annual household income; Model 2: model 1 plus smoking status, drinking status, and physical activity; Model 3: model 2 plus body mass index, history of diabetes mellitus and hypertension, and family history of heart attack. BMI indicates body mass index; HR, hazard ratio; MD, major depression.

a *P* values for interaction were assessed by including an interaction term of MD and corresponding stratified factor in model 3.

Geographic location modified the association between MD and risk of IHD (*P* for interaction=0.005; Table 2): MD was associated with risk of IHD in urban (HR 1.72, 95% CI 1.39–2.14) but not rural residents (HR 1.13, 95% CI 0.93–1.37) (Table 2). The association between MD and risk of IHD was not modified by sex (*P* for interaction=0.23), age (*P* for interaction=0.10), or BMI (*P* for interaction=0.98).

The sensitivity analysis, after excluding incident IHD cases diagnosed within the first 2 years of follow‐up, showed that the positive association did not change (HR 1.29, 95% CI 1.10–1.52) (Table 2). When the effect of severity of depression on the risk of IHD was assessed, we found that the HR (95% CI) of IHD was 1.13 (1.04–1.23) for those with depressive symptoms only and 1.33 (1.15–1.53) for those with MD versus those without depressive symptoms (Table 3).

**Table 3:** Depressive Symptoms With/Without Major Depression (MD) and Risk of Incident Ischemic Heart Disease^a^

	No Depressive Symptoms	Depressive Symptoms Only	MD
Cases/person‐years	23 947/3 323 751	568/78 110	190/21 681
Model 1	1.00	1.11 (1.02–1.21)	1.32 (1.15–1.53)
Model 2	1.00	1.12 (1.03–1.22)	1.31 (1.13–1.51)
Model 3	1.00	1.13 (1.04–1.23)	1.33 (1.15–1.53)

Model 1: adjusted for age, sex, geographic location, marital status, education, and annual household income; Model 2: model 1 plus smoking status, drinking status, and physical activity; Model 3: model 2 plus body mass index, history of diabetes mellitus and hypertension, and family history of heart attack.

a Data are shown as hazard ratio (95% CI).

## Discussion

In this large‐scale prospective cohort study, we found that past year MD was associated with a 32% higher risk of developing IHD among Chinese adults aged 30 to 79 years. The association was independent of sociodemographic characteristics, family history, lifestyle factors, and baseline comorbidities (obesity, diabetes mellitus, and hypertension). In addition, the association was more evident in urban than rural residents.

In our study, we have found a statistically significant association between MD and risk of IHD with a multivariable‐adjusted HR of 1.32. The result is consistent with the finding from a systematic review of 30 cohort studies that reported a pooled relative risk of 1.30 (95% CI 1.22–1.40).^7^ In this review, most studies were conducted in the United States (n=15) or European countries (n=12), while only 2 studies were in Asians: 1 in Chinese Taiwan and the other in Hong Kong. The study population in Chinese Taiwan^10^ comprised clinically diagnosed depression patients (n=7937) and nondepressed individuals (n=31 748) selected from an insurance database in a follow‐up to 9 years. Coronary events (myocardial infarction, percutaneous coronary intervention, and coronary artery bypass grafting) were used as study outcomes. After controlling for established cardiovascular risk factors, depression was associated with a 38% increased risk of coronary events (HR 1.38; 95% CI 1.19–1.60). In the study among elderly Chinese in Hong Kong,^11^ depressive symptoms were screened using the 15‐item Geriatric Depression Scale among 21 473 men and 41 366 women at Elderly Health Centers. Incident nonfatal CHD cases were not available, and CHD mortality was assessed as the outcome. A positive association was reported between depressive symptoms and CHD mortality in men (HR 1.41; 95% CI 1.08–1.84) but not women (HR 0.94; 95% CI 0.75–1.16). Therefore, our study is the first on this topic in mainland China using a community‐based population from 10 regions across China. We also found that the association was stronger in men compared to that in women (HR 1.50 versus 1.26), although there was no significant effect modification (*P* for interaction=0.23), which may be attributable to the small number of depressed individuals. The difference between men and women was also consistent with the overall finding from the systematic review, in which the pooled HR was 1.38 among men versus 1.17 among women.^7^

With a sample size of about 0.5 million, our study has been the largest one on this research topic so far. We analyzed data from 486 541 participants with 24 705 incident IHD cases during a median follow‐up of 7.2 years. The systematic review of 30 prospective cohort studies included a total of 893 850 participants with 59 062 CHD cases.^7^ The largest original study prior to our investigation used data from the US Veterans Administration electronic medical records and included 345 949 men and women aged 25 to 80 years, in which the authors reported that depression was associated with a 29% increased risk of myocardial infarction.^16^ Therefore, our main finding was consistent with that from the other large cohort studies, and provides strong evidence from Chinese populations that depression is an independent risk factor for heart disease.

Most previous studies used self‐reported questionnaires to screen for depressive symptoms and only a few used clinical diagnosis of depression as the exposure. There is controversy over whether the association between depression and risk of IHD would differ when depression was identified using self‐reported scales and structured clinical diagnostic interviews. Such controversy was reflected in the contrasting results from 2 previous meta‐analyses.^7^, ^8^ In our study, depression was screened by a few screening questions, followed by the diagnostic Composite International Diagnostic Interview‐Short Form. We compared individuals who responded positively to screening questions but did not meet the diagnostic criteria (depressive symptoms only) with those who had no depressive symptoms. Although statistically significant, the effect size (HR 1.13) for the association between depressive symptoms and IHD risk was much lower than that for MD. It suggests that more severe depression bears a higher risk of IHD and the dose–response relationship may exist. Although the current analysis revealed that MD was significantly more prevalent in rural areas than in urban areas (0.71% versus 0.48%), the multivariable‐adjusted MD–IHD association was statistically significant in urban but not rural residents. A previous study in 4 provinces of China also found a higher prevalence of depressive disorders in rural compared to urban residents.^17^ The exact reasons for the urban–rural difference in the prevalence of depression, as well as the association between depression and IHD were unclear. No study has specifically examined this issue yet, and we speculated that the stress of living in an urban setting might enhance the impact of MD on IHD risk. However, more studies are needed to validate the urban–rural differences and explore the potential reasons.

There are several potential mechanisms through which depression confers excess risk of IHD, with pathophysiological change and unhealthy behaviors as the major ones. On the one hand, as summarized in previous reviews,^18^, ^19^ depression can lead to hyperactivity of the hypothalamic–pituitary–adrenal axis, lower heart rate variability, greater catecholamine levels, platelet activation, and inflammatory processes, which are common contributors to the development of IHD. On the other hand, unhealthy behaviors (smoking, physical inactivity, unhealthy diet, dangerous alcohol use, and lack of medication adherence, etc)^18^, ^20^ and obesity^21^ were more common among depressed patients. Interestingly, participants with MD in our study were less likely to be smokers and had a slightly lower BMI compared to participants without MD at baseline. This might be because of the fact that women were more likely to be depressed while less likely to be smokers. When stratified by sex, depressed participants were more likely to be current smokers in both men and women (data not shown). However, BMI remained lower in people with MD compared to those without MD in both sexes (data not shown), which has been consistently observed in previous studies in Chinese and Korean populations, suggesting the “jolly fat” hypothesis.^22^, ^23^, ^24^, ^25^ Furthermore, depression is common in people with chronic diseases, as evidenced by a higher prevalence of MD in people with diabetes mellitus in our study. We have adjusted for baseline comorbidities and various behavior factors in the model and the results remained unchanged, although residual confounding because of lack of information for other potential factors (such as other comorbidities related to both depression and IHD, poor diet, and lack of medication adherence) was still possible.

Our study has the strengths of large sample size, a relatively long follow‐up, standardized measure of depression by the structured diagnostic interviews, well‐documented IHD incidence, and adjustment for multiple potential confounders. However, some limitations still need to be acknowledged. First, the 12‐month prevalence of MD detected by Composite International Diagnostic Interview‐Short Form in our study (0.61%) was quite low compared to findings from previous studies in Western and Chinese populations. For example, the US National Comorbidity Survey Replication in a targeted population aged 18 years or older reported a 12‐month prevalence of 6.6%^26^; a previous study in 4 provinces of China found that the adjusted 1‐month prevalence of major depressive disorder was 2.1%^17^; and in the World Health Organization World Mental Health Surveys, the 1‐year prevalence of major depressive episode was 1.8% for adults aged 18 years and older in Beijing and Shanghai.^27^ The differences in prevalence across countries and studies could partly be attributable to different tools used to assess MD, different study populations, or cultural backgrounds. For example, the previous study in 4 provinces of China detected MD through a semistructured interview administered by psychiatric nurses,^17^ which allowed them to rephrase the screening questions according to local dialect, while ours was a fully structured assessment performed by health workers. In addition, health workers' ability to detect depression may not be as optimal as psychiatric nurses' ability. In specific cultural context, Chinese adults may deem MD a stigma and deny their mental condition or experience MD in a nontraditional way.^28^ Furthermore, the current study only recruited those who volunteered to participate, while more depressed patients would be less likely to be included because of their loss of interest in most things. All of the factors mentioned above may eventually lead to the low detection rate of MD. The impact of the relatively low prevalence of depression in our study on the association between MD and risk of IHD remains unclear. On the one hand, more severe cases could be easier to be screened and detected in population‐based studies and a stronger MD–IHD association could be observed; on the other hand, severely depressed individuals may not be willing to participate in a long‐term follow‐up study, and a weaker MD–IHD association may be expected. Therefore, a validation study of sensitivity and specificity of the instrument used in our study against clinical diagnosis is preferred, but unfortunately this is not currently available.

Second, information about the change and duration of depression status during the follow‐up was not available in our study. Third, although we have adjusted for many cardiovascular risk factors in the model, residual confounding may still remain. Finally, we were unable to distinguish fatal and nonfatal IHD in our analysis because of data limits, although differences in risk estimates for fatal and nonfatal events have been shown to be nonsignificant.^9^

## Conclusions

In conclusion, we found a 32% higher risk of developing IHD for participants with baseline MD in a mega‐cohort of about 0.5 million Chinese adults, independent of other major cardiovascular risk factors. Future studies might direct attention to potential strategies to prevent IHD among people with depression, such as cognitive behavioral therapy for stress management and stigma reduction,^29^ or preventive measures for modifiable risk factors for IHD.

## Appendix

### China Kadoorie Biobank Collaborative Group

(a) International Steering Committee

Liming Li, Zhengming Chen, Junshi Chen, Rory Collins, Richard Peto.

(b) Study Coordinating Centers

International (ICC, Oxford): Zhengming Chen, Garry Lancaster, Xiaoming Yang, Alex Williams, Margaret Smith, Ling Yang, Yumei Chang.

National (NCC, Beijing): Yu Guo, Guoqing Zhao, Zheng Bian, Lixue Wu, Can Hou.

### Regional (RCC, 10 Areas in China)

Qingdao

Qingdao CDC: Zengchang Pang, Shaojie Wang, Yun Zhang, Kui Zhang.

Licang CDC: Silu Liu.

Heilongjiang

Provincial CDC: Zhonghou Zhao, Shumei Liu, Zhigang Pang.

Nangang CDC: Weijia Feng, Shuling Wu, Liqiu Yang, Huili Han, Hui He.

Hainan

Provincial CDC: Xianhai Pan, Shanqing Wang, Hongmei Wang.

Meilan CDC: Xinhua Hao, Chunxing Chen, Shuxiong Lin.

Jiangsu

Provincial CDC: Xiaoshu Hu, Minghao Zhou, Ming Wu.

Suzhou CDC: Yeyuan Wang, Yihe Hu, Liangcai Ma, Renxian Zhou, Guanqun Xu.

Guangxi

Provincial CDC: Baiqing Dong, Naying Chen, Ying Huang.

Liuzhou CDC: Mingqiang Li, Jinhuai Meng, Zhigao Gan, Jiujiu Xu, Yun Liu.

Sichuan

Provincial CDC: Xianping Wu, Yali Gao, Ningmei Zhang.

Pengzhou CDC: Guojin Luo, Xiangsan Que, Xiaofang Chen.

Gansu

Provincial CDC: Pengfei Ge, Jian He, Xiaolan Ren.

Maiji CDC: Hui Zhang, Enke Mao, Guanzhong Li, Zhongxiao Li, Jun He.

Henan

Provincial CDC: Guohua Liu, Baoyu Zhu, Gang Zhou, Shixian Feng.

Huixian CDC: Yulian Gao, Tianyou He, Li Jiang, Jianhua Qin, Huarong Sun.

Zhejiang

Provincial CDC: Liqun Liu, Min Yu, Yaping Chen.

Tongxiang CDC: Zhixiang Hu, Jianjin Hu, Yijian Qian, Zhiying Wu, Lingli Chen.

Hunan

Provincial CDC: Wen Liu, Guangchun Li, Huilin Liu.

Liuyang CDC: Xiangquan Long, Youping Xiong, Zhongwen Tan, Xuqiu Xie, Yunfang Peng.

## Sources of Funding

This work was supported by grants (81390541, 81390542, 81202266, and 81230069) from the National Natural Science Foundation of China. The CKB baseline survey and the first re‐survey were supported by a grant from the Kadoorie Charitable Foundation in Hong Kong. The long‐term follow‐up is supported by grants from the UK Wellcome Trust (088158/Z/09/Z, 104085/Z/14/Z), and by a grant from the Chinese Ministry of Science and Technology (2011BAI09B01). In addition, the British Heart Foundation, UK Medical Research Council, and Cancer Research UK also provided core funding to the Clinical Trial Service Unit and Epidemiological Studies Unit (CTSU) at Oxford University. The funders had no role in the study design, data collection, data analysis and interpretation, writing of the report, or the decision to submit the article for publication.

## Disclosures

None.
